# Needle-free atrial transseptal access: A safe and reproducible alternative for left atrial catheterization

**DOI:** 10.1016/j.hroo.2024.09.005

**Published:** 2024-09-18

**Authors:** Alex D. Pacheco-Bouthillier, Jorge Javier Miguel-González, Adriana González-Martínez, Anna G. Everding-Rodríguez, Silvia S. Gómez-Delgadillo, Ángel E. Chávez-Torres, Angélica Fregoso-Sánchez, Benigno Ferreira-Piña, Óscar S. Lomelí-Sánchez, Hugo E. Coutiño-Moreno, Vitelio A. Mariona-Montero

**Affiliations:** 1 Departamento de Investigación, Instituto Cardiovascular de Mínima Invasión, Zapopan, México; 2 Unidad de Electrofisiología, Hospital de Cardiología, Centro Médico Nacional Siglo XXI, Instituto Mexicano del Seguro Social, Mexico City, México; 3 Unidad de Cardiología, Hospital Civil Fray Antonio Alcalde, Guadalajara, Mexico; 4 Unidad Médica de Alta Especialidad en Pediatría, Centro Médico Nacional de Occidente, Instituto Mexicano del Seguro Social, Guadalajara, México

**Keywords:** Transseptal catheterization, Left heart catheterization, Ablation, Cardiac arrhythmia, Atrial septum

## Abstract

**Background:**

Left atrial catheterization is a common procedure in electrophysiology labs to treat arrhythmogenic substrates on the left side of the heart. Needle transseptal puncture is the standard approach, but it can lead to complications related to device design or operator technique. To reduce these complications, needle-free alternatives have been explored.

**Objective:**

This study aims to report the first multicenter application of the needle-free transseptal access (NeFTA) approach, assessing its safety and efficacy in patients undergoing electrophysiological procedures.

**Methods:**

This retrospective, observational multicenter study evaluated the safety and efficacy of the NeFTA approach in patients undergoing ablation of left arrhythmogenic substrates across 3 electrophysiology centers in Mexico. NeFTA uses only a guidewire, without a needle or sharp guidewire. The sheath, with a dilator, is guided into the fossa ovalis under fluoroscopic guidance, using anterior force and clockwise torque to allow the guidewire to puncture the septum with minimal risk.

**Results:**

The NeFTA technique was used in 366 patients. Most sheaths were deflectable, with nondeflectable sheaths in 43.4% of cases. Left atrial access via NeFTA was successful in 96.18% of cases, with pericardial effusion as the only complication (0.55% rate).

**Conclusion:**

This technique was reproducible and safe, achieving successful access on the first or second attempt in 96.18% of cases, regardless of the operator.

## Graphical abstract

**Figure:**
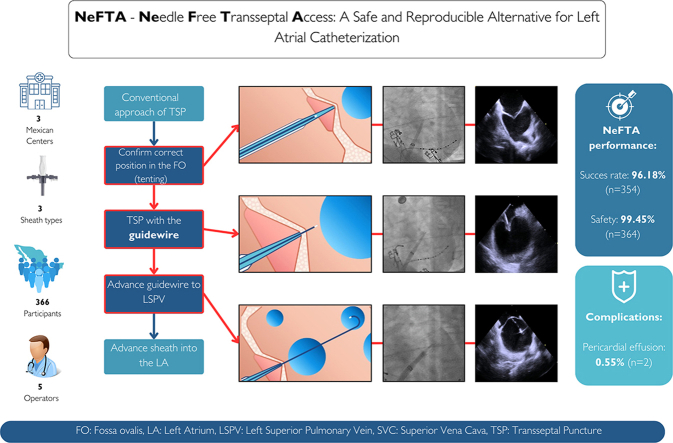

**Key Findings**

▪ The needle-free transseptal access (NeFTA) approach achieved successful left atrial access in 96.18% of cases, demonstrating its efficacy in electrophysiological procedures.
▪ The only reported complication was pericardial effusion, occurring in just 0.55% of cases, indicating a favorable safety profile for the NeFTA technique.
▪ The NeFTA technique simplifies the transseptal access process by using a high-support guidewire with the cryoballoon sheath, eliminating the need for multiple sheath exchanges and reducing overall procedure duration. This approach achieved a 97.3% success rate in cryoablation procedures, further supporting its efficiency.
▪ The NeFTA technique's demonstrated safety using only fluoroscopy is particularly valuable in settings in which access to advanced imaging technologies, such as transesophageal echocardiography and 3-dimensional mapping, is limited. This makes the technique more accessible and practical in resource-constrained environments.
▪ The NeFTA approach may offer cost advantages by eliminating the need for additional equipment, potentially making it more cost-effective than traditional needle transseptal puncture and radiofrequency puncture techniques.

## Introduction

Left atrial (LA) catheterization has become a standard procedure in electrophysiology laboratories due to the high prevalence of arrhythmogenic substrates on the left side of the heart, such as atrial fibrillation, atrial flutter, and left accessory pathways, that require treatment. It is therefore imperative to improve techniques for more efficient and safe access to the LA. Over time, numerous techniques have been proposed for performing left heart catheterization. These include transbronchial puncture, percutaneous atrial puncture, arterial retrograde catheterization, and the currently most accepted approach, needle transseptal puncture (N-TSP).^1^

It is thus imperative for interventional cardiac electrophysiologists to possess a comprehensive understanding of the anatomy of the interatrial septum and adjacent structures. The atrial septum has an average anatomical size of 141 mm^2^ in children and 890 mm^2^ in adults. The fossa ovalis accounts for 26% and 28% of the total area of the interatrial septum, respectively.^2^ The fossa ovalis serves as the primary site for transseptal access due to its thinner than average thickness in comparison to the surrounding region, which facilitates entry into the LA.^3^

For a considerable period of time, N-TSP has been the prevailing methodology for TSP, utilizing a sharp needle within a sheath, with notable efficacy. Nevertheless, complications have been documented, predominantly associated with the device design or the operator's ability to manipulate the sheath. Such complications include cardiac perforation, which may result in cardiac tamponade, as well as aorta perforation, which can have significant hemodynamic implications. Correcting these issues often demands the implementation of advanced and complex therapeutic modalities.^4^, ^5^, ^6^, ^7^

The N-TSP approach has been described in detail in numerous publications in the scientific literature. While this technique offers several advantages, it is not without limitations. In certain instances, the presence of a resistant atrial septum may render the application of mechanical pressure from the needle insufficient to perforate the septum. For this reason, modifications to the technique have been developed in which radiofrequency (RF) is used at the tip of the needle or directly to the guidewire to perforate the septum.^8^, ^9^, ^10^, ^11^ This modification to the conventional procedure has gained popularity in recent years, particularly in cases in which the N-TSP approach was unsuccessful or in cases in which a second puncture was necessary. While this modification has been demonstrated to be effective,^12^^,^^13^ concerns regarding safety remain, as the introduction of RF through the needle or guidewire could potentially result in further complications.

The search for a needle-free alternative was initiated as a means of reducing complications associated with N-TSP. A tapered 0.014′′ diameter nitinol wire with a sharp distal tip and a J-shape has recently been reported as a safe option that avoids common complications.^14^

The initial report of a genuine needle-free alternative was a modification of the conventional technique. This approach exclusively depended on the guidewire to carry out the TSP and employed a 0.032′′ J-tipped guidewire guided by intracardiac ultrasound and fluoroscopy to puncture the interatrial septum.^15^

The objective of this study is to present the initial multicenter application of the needle-free transseptal access (NeFTA) approach. NeFTA represents a further adaptation of the needle-free approach. This technique employs either a conventional or high-support guidewire for transseptal access, without the use of a needle or sharp guidewire within the sheath. The study presents a retrospective analysis of a consecutive group of patients who underwent NeFTA treatment for electrophysiological indications at 3 different centers in Mexico.

## Methods

### Patients

This retrospective, observational, multicenter study analyzed a cohort of patients who underwent electrophysiological procedures to ablate left arrhythmogenic substrates with the NeFTA approach for TSP in a consecutive manner. This study was conducted at 3 interventional cardiac electrophysiology centers in Mexico: Instituto Cardiovascular de Mínima Invasión, Hospital de Cardiología del Centro Médico Siglo XXI, and Unidad Médica de Alta Especialidad en Pediatría del Centro Médico Nacional de Occidente. It spanned a period of over 6 years. The research reported in this paper adhered to the Helsinki Declaration. Mandatory permissions for retrospective data management were granted by the participating institutions. All patients received a verbal explanation of the procedure, with informed consent obtained from each patient and/or their parent/guardian. As this study utilized de-identified, retrospective data, a waiver of additional consent was granted by the institutional review board.

### Operator training

The NeFTA technique was carried out by a variety of operators, several of whom lacked experience in TSP within any particular technique (Table 1). All cases were executed or overseen by 1 of the 2 highly experienced operators in the N-TSP approach (A.D.P.B. and V.A.M.M.).

**Table 1:** Operator characteristics

Operator	Grade	Experience in N-TSP
0	Chief consultant electrophysiologist	Highly experienced
1	Chief consultant electrophysiologist	Highly experienced
2	Consultant electrophysiologist	Experienced
3	Interventional electrophysiology fellow	Without previous experience
4	Interventional electrophysiology fellow	Without previous experience

This table outlines the operators involved in executing the NeFTA technique, highlighting their level of experience in N-TSP.

N-TSP = needle transseptal puncture; NeFTA = needle-free transseptal access.

### Needle-free transseptal access technique

After accessing the femoral vein, the metallic guidewire included in the sheath system pack (super stiff 0.032′′, 180 cm; Abbott) is advanced toward the superior vena cava and the innominate vein, with the J-shaped tip facing in a forward direction. If a cryoballoon sheath (CBS) is utilized, the identical protocol is executed along with a high-support guidewire (Amplatz super stiff 0.035′′, 260 cm; Boston Scientific). The sheath is advanced over the guidewire, positioning the dilator's tip toward the innominate vein. The guidewire is then fully withdrawn inside the dilator, leaving no lumen space. If a deflectable sheath (DS) is used, flexion of the sheath must be performed in a left anterior oblique view.

Afterward, the entire system is retracted within the same frame of reference to observe the descent of the tip of the sheath into the right atrium and then into the fossa ovalis. At this stage, as will be done using the N-TSP, it is crucial to confirm the position of the dilator's tip in relation to the reference catheters, such as the coronary sinus (CS) and His catheters. In a left anterior oblique view, it is imperative that the dilator's tip runs parallel to the CS catheter and extends beyond the His catheter, positioning itself posteriorly. In the right anterior oblique view, it should be observed once again in parallel with the CS catheter and behind the His catheter.

If it is necessary to verify the position, contrast of the dilator will have to be administered to observe the typical distention of the septum. However, it is noteworthy that this process necessitates fully retracting the guidewire from the dilator prior to administering the contrast and subsequently adjusting the guidewire until it reaches the tip of the dilator once again.

**Figure 1:**
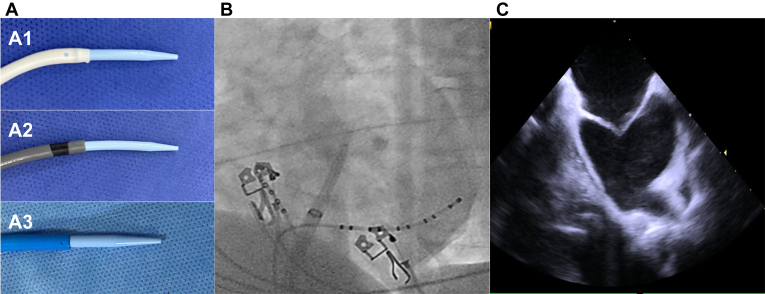
Confirming the position of the sheath. A: Sheaths with the wire at the tip of the dilator: nondeflectable sheath (A1), deflectable sheath (A2), cryoballoon sheath (A3). B: Fluoroscopy view perpendicular to the mitral valve with the sheath parallel to it. C: Intracardiac echocardiography showing the system being pushed toward the atrial septum, with satisfactory distension of the fossa ovalis.

At this moment, the system is being pushed forward, resulting in an anterior force acting upon it. As a result, the operator must apply a clockwise torque to maintain the sheath's alignment with the reference catheters. One approach could be to observe the rings of both, the CS catheter, and the distal tip of the sheath as a solid piece, rather than as a circle. By following this process, it is possible to ensure that the fluoroscopic view is truly perpendicular to the mitral valve plane and that the sheath is parallel to it (Figure 1).

While pushing the system forward, it is common to experience dilator tip flexion. If the degree of flexion is insignificant, the procedure can proceed; however, if it is significant, it is recommended to dismantle the entire system and slide the sheath in a manner that preserves the position of the dilator's tip without losing alignment. More tension can be exerted on the fossa ovalis by pushing the system further forward.

**Figure 2:**
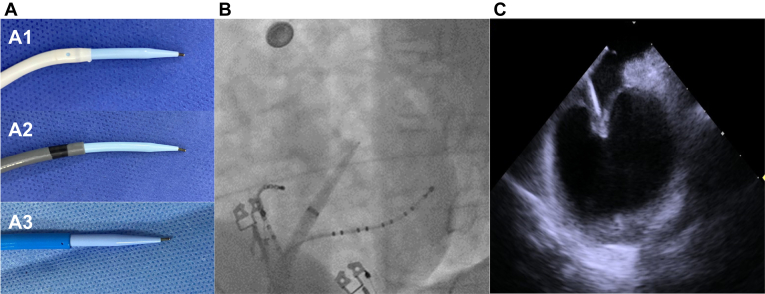
Performing the transseptal puncture. A: Sheaths with the J-tip of the wire positioned at the front passing through the atrial septum: nondeflectable sheath (A1), deflectable sheath (A2), cryoballoon sheath (A3). B: Fluoroscopy view of the J-tip passing through the atrial septum. C: Intracardiac echocardiography showing the J-tip passing through the atrial septum.

It is crucial to keep in mind that performing the NeFTA technique becomes easier when it is feasible to maintain satisfactory positioning and distension of the fossa ovalis. Once both these factors are confirmed, the TSP process should be conducted using the guidewire (J-tip positioned at the front) with a quick but forceful movement, passing through the septum with the same tactile sensation as with regular puncture (Figure 2). After TSP, heparin will be given to maintain an activated clotting time of about 300 to 400 seconds as in the conventional workflow.

**Figure 3:**
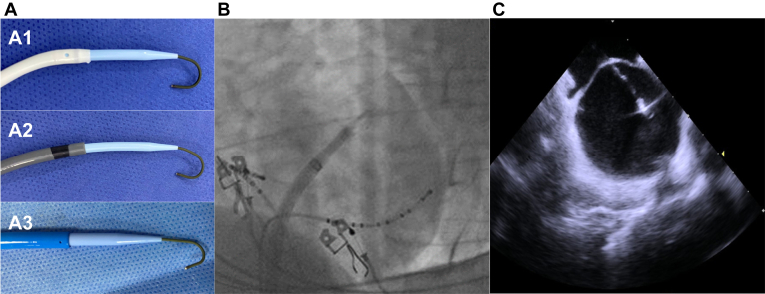
The tip of the guidewire regains its J-shape upon crossing the septum. A: Sheaths with the guidewire regaining the J-shape: nondeflectable sheath (A1), deflectable sheath (A2), cryoballoon sheath (A3). B: Fluoroscopy view of the guidewire crossing the septum regaining its J-shape. C: Intracardiac echocardiography showing the guidewire crossing the septum regaining its J-shape.

The tip of the guidewire regains its J-shape upon crossing the septum, significantly reducing the risk of perforation (Figure 3). Subsequently, the operator may guide the guidewire toward the left or right superior pulmonary vein and shift the system across the septum.

If the system had to be disassembled during the process, it must be reassembled before being introduced into the LA. Subsequently, the dilator and guidewire are removed, and the sheath is washed conventionally to continue the procedure.

### Data collection and statistical analysis

The success of the technique was determined by the achievement of TSP with the NeFTA approach within the initial 2 attempts. In the event of N-TSP being required, the technique was deemed unsuccessful. Only complications directly attributable to the puncture were assessed. The assessment of the technique was conducted without regard to the particular type of sheath used.

## Results

The NeFTA technique was employed on 366 patients, who were subsequently included within this study. The average age was 35 (range 4–77) years. Of these patients, 64.42% (n = 239) were male. Wolff-Parkinson-White syndrome was the most observed pathology (n = 147 [39.62%]), followed by atrial fibrillation (n = 121 [32.61%]) (Table 2). Of all the sheaths used, 55.3% (n = 205) were DSs, of which 65 (31.7%) were CBS. Nondeflectable sheaths (NDSs) were used in 161 procedures, accounting for 43.4% of cases. LA access via the NeFTA technique was successful in 96.18% of instances (n = 352).

**Table 2:** Characteristics of patients undergoing the NeFTA technique

	N	%
Age		
4–11 y	46	12.40
11–18 y	80	21.56
18–25 y	24	6.47
25–32 y	20	5.39
32–39 y	30	8.09
39–46 y	37	9.97
46–53 y	34	9.16
53–60 y	42	11.32
60–67 y	38	10.24
67–74 y	11	2.96
74–81 y	4	1.08
Biological sex assigned at birth		
Female	127	34.23
Male	239	64.42
Diagnosis		
Wolff-Parkinson-White	147	39.62
Atrial fibrillation	121	32.61
Hidden accessory pathway	42	11.32
Atrial tachycardia	24	6.47
Ventricular tachycardia	15	4.04
Ventricular extrasystole	6	1.62
Atrial flutter	6	1.62
Hypertrophic cardiomyopathy	4	1.08
Cardioinhibitory syncope	1	0.27

NeFTA = needle-free transseptal access.

Of the 12 patients in which the puncture was unsuccessful, the mean age was 43.3 (range 14–76) years, 88% were male patients, atrial fibrillation and Wolff-Parkinson-White syndrome were the most common diagnoses, and 75% of the punctures were performed with an NDS. Unsuccessful punctures were present at any point of the operators learning curve (Table 3).

**Table 3:** Characteristics of patients in whom the NeFTA technique was unsuccessful

Patient	Age (y)	Sex	Diagnostic	Sheath type	Operator	Puncture number
1	65	M	VT	NDS	0	9/154
2	15	M	WPW	NDS	3	1/54
3	14	M	WPW	NDS	0	23/154
4	42	M	AF	CBS	3	16/54
5	14	M	WPW	NDS	3	44/54
6	44	M	VT	NDS	4	15/52
7	64	F	WPW	NDS	3	47/54
8	48	M	AF	NDS	4	24/52
9	30	M	AF	NDS	4	27/52
10	61	M	AF	NDS	4	48/52
11	76	M	AF	DS	0	154/154
12	47	F	AF	DS	1	95/95

Values are n/n, unless otherwise indicated. This table includes diagnosis, sheath type, operator, and time of unsuccessful puncture for each operator learning curve.

AF = atrial fibrillation; CBS = cryoballoon sheath; DS = deflectable sheath; F = female; M = male; NDS = nondeflectable sheath; NeFTA = needle-free transseptal access; VT = ventricular tachycardia; WPW = Wolff-Parkinson-White.

The sole associated complication was pericardial effusion (n = 2 [0.55%]). The complications related to the puncture had no significant hemodynamic implications and no further interventions were necessary. However, both procedures were cancelled and rescheduled. The same operator experienced both incidents in the first phase of his learning curve, and they were performed with an NDS (Table 4).

**Table 4:** Characteristics of patients who experienced complications due to the NeFTA technique

Patient	Age	Sex	Diagnostic	Sheath type	Operator	Puncture number
1	26	F	WPW	NDS	4	3/52
2	25	F	VT	NDS	4	13/52

Values are n/n, unless otherwise indicated. This table includes diagnosis, sheath type, and complication time for the operator learning curve.

F = female; NDS = nondeflectable sheath; NeFTA = needle-free transseptal access; VT = ventricular tachycardia; WPW = Wolff-Parkinson-White.

Fluoroscopic guidance was used in 100% of the patients. Intracardiac or transesophageal echocardiography was used only if the complexity of the ablation procedure required them (n = 14 [3.8%]).

## Discussion

### The efficacy and reproducibility of the NeFTA technique

This study presents the most comprehensive patient series to date for TSP using the NeFTA approach with diverse sheath types. In their 2015 study, Giudici and colleagues^15^ initially described the technique and proposed it as a safe and straightforward alternative to N-TSP. The procedure does not require the use of needles and is performed using NDSs. Of the 100 patients included in Giudici and colleagues’ study, 99 achieved successful punctures, and no complications were associated with the use of the technique. The electrophysiologists in this study successfully replicated and enhanced their technique using DS, CBS, and NDS on 366 patients. They demonstrated the safety and effectiveness of the technique with a success rate of 96.18% (n = 352) and a complication rate of <1% (0.55% [n = 2]).

### Complications

In contrast to the N-TSP approach, performing the puncture exclusively with the guidewire reduces the number of steps and exchanges required by the operator to reach the LA. This results in a reduction in time and an associated reduction in the risk of access complications, air emboli, and thrombus formation.^15^ In a study conducted by Matoshvili and colleagues,^16^ a total of 4690 procedures that required N-TSP were analyzed. A total of 48 (1.2%) complications were observed, and the authors hypothesized that operator experience was a significant factor in reducing the overall complication rate. The sole complication recorded in association with the puncture was pericardial effusion, occurring in 2 (0.55%) cases. The analysis indicates that these occurrences were the result of the operator's inexperience at the outset of their learning curve (see Table 4). It is important to note, however, that this study has a significant limitation in that it is not a randomized controlled trial comparing the NeFTA technique with conventional N-TSP. Therefore, further research is required.

### Imaging tools

A variety of imaging tools are employed to facilitate the TSP process. Specifically, transesophageal echocardiography has been shown to enhance the safety of punctures.^17^ Moreover, 3-dimensional mapping is facilitating the implementation of fluoroscopy-free and echocardiography-free techniques.^18^ In some countries, obtaining access to imaging technologies may present certain challenges. In light of these considerations, the development of a technique that demonstrates safety using only fluoroscopy has significant implications for our context.

### Use of a deflectable sheath vs a nondeflectable sheath as a distinguishing factor

The data indicate a correlation between sheath type and overall technique performance. An NDS was utilized in 75% of unsuccessful punctures (Table 3) and 100% of complications (Table 4). It is also noteworthy that technique failures occurred at different points in each operator's learning curve (Table 3), while complications occurred exclusively at the initial stage (Table 4). It is our hypothesis that the deflectable sheath offers a technical advantage, allowing for easier access to sites of interest, irrespective of the operator's experience.

### Application of the NeFTA technique in the pediatric population

A study conducted by Koca and colleagues^19^ on a pediatric population included 45 patients with a weight of <30 kg who underwent N-TSP. The incidence of complications was 2.2%. Our study also evaluated punctures performed on a pediatric population, with 30.32% of patients under the age of 15 years (n = 111) and 4.6% under the age of 8 years (n = 17). A successful puncture was achieved in 97.29% (n = 108) of cases. The absence of complications in this patient cohort provides evidence that the NeFTA approach is both safe and practical in pediatric patients. Nevertheless, further research is required.

### Zero-exchange approach in cryoballoon procedures

**Figure 4:**
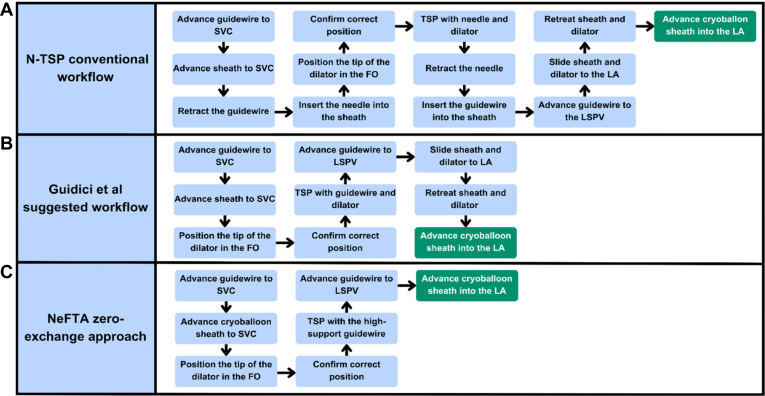
Three different cryoablation transseptal puncture techniques: sequencing steps compared. A: Needle transseptal puncture convential workflow (13 steps). B: Guidici and colleagues^15^ suggested workflow to gain access with a nondeflectable sheath before switching to the cryoballoon sheath (9 steps). C: Needle-free transseptal access (NeFTA) zero-exchange approach in which access is obtained directly with the cryoballoon sheath (7 steps). FO = fossa ovalis; LA = left atrium; LSPV = left superior pulmonary vein; N-TSP = needle transseptal puncture; SVC = superior vena cava.

In contrast to the conventional N-TSP approach (Figure 4A) and the recommendations of Giudici and colleagues^15^ to use an NDS initially when using larger sheaths, such as CBS, and to leave the guidewire in the left superior pulmonary vein before changing the entire system (Figure 4B), the NeFTA technique is executed using the CBS, utilizing a high-support guidewire to implement the puncture, thus eliminating steps and sheath exchanges to gain access to the LA (Figure 4C). The NeFTA technique was employed in cryoablation procedures in 16.6% (n = 57) of patients, resulting in a success rate of 97.3% (n = 56). This results in a notable reduction in the number of access manipulations, material exchanges, and procedure duration.

A comparable scenario is presented in the study conducted by Yap and colleagues,^20^ which evaluated the efficacy of an innovative integrated needle within the dilator for TSP in the CBS. The findings indicate that the use of this integrated needle resulted in a substantial reduction in the duration of the procedure, with zero changes to the sheath systems. A comparable result was observed in the study conducted by Khaykin and colleagues,^10^ in which the puncture was performed using direct-wire RF electrocautery. A cost-effective analysis conducted by Sanchez and colleagues^21^ comparing N-STP and RF punctures revealed that the RF puncture is more cost-effective than N-TSP in probabilistic sensitivity analyses despite higher equipment costs. Not only is the outcome theoretically equally efficient and safe when employing the NeFTA approach, but also it is worthwhile to consider the advantage of eliminating additional equipment costs. However, a dedicated cost-effectiveness analysis is necessary for verification.

### Other needle-free alternatives

In recent years, alternative solutions to the needleless TSP have been developed. A 0.014′′ nitinol guidewire with a sharp, preformed J-shaped tip is capable of piercing the interatrial septum and restoring its atraumatic shape once it has been inserted into the LA. A multicenter study by Chow and colleagues^14^ reported a 100% success rate with no reported complications in a sample of 145 patients. The NeFTA technique also aims to avoid needles and enter the LA atraumatically. This could theoretically reduce complication rates; however, further research is required to ascertain whether the absence of a needle in the NeFTA approach could influence the overall success of the puncture in specific patients.

### Limitations

This study has limitations inherent to its retrospective, observational design. First, the lack of a control group prevents a direct comparison of the safety and efficacy of the NeFTA approach to other traditional methods. As such, it is challenging to draw definitive conclusions about whether the NeFTA technique improves success rates or reduces complications compared with the N-TSP. Additionally, the retrospective nature of the study may introduce selection bias, as patient data were collected based on existing medical records without randomization or blinding. Future prospective studies with randomized control groups are needed to validate these findings and to better understand the potential advantages and limitations of the NeFTA technique in comparison to traditional methods.

## Conclusion

This technique has been demonstrated to be both reproducible and safe, resulting in success on the first or second attempt, regardless of the operator, in 96.18% of cases, with a complication rate of <1%.
